# The ciliary marginal zone of the zebrafish retina: clonal and time-lapse analysis of a continuously growing tissue

**DOI:** 10.1242/dev.133314

**Published:** 2016-04-01

**Authors:** Yinan Wan, Alexandra D. Almeida, Steffen Rulands, Naima Chalour, Leila Muresan, Yunmin Wu, Benjamin D. Simons, Jie He, William A. Harris

**Affiliations:** 1 Department of Physiology, Development and Neuroscience, University of Cambridge, Cambridge CB2 3DY, UK; 2 Howard Hughes Medical Institute, Janelia Research Campus, Ashburn, VA 20147, USA; 3 Cavendish Laboratory, Department of Physics, J.J. Thomson Avenue, University of Cambridge, Cambridge CB3 0HE, UK; 4 Wellcome Trust/Cancer Research UK Gurdon Institute, University of Cambridge, Tennis Court Road, Cambridge CB2 1QN, UK; 5 Wellcome Trust-Medical Research Council Stem Cell Institute, University of Cambridge, Tennis Court Road, Cambridge CB2 1QR, UK; 6 Institute of Neuroscience, Chinese Academy of Sciences, Shanghai 200031, China

**Keywords:** Stem cells, Progenitor cells, Retina, Ciliary marginal zone, Live imaging, Clonal analysis, Zebrafish

## Abstract

Clonal analysis is helping us understand the dynamics of cell replacement in homeostatic adult tissues (Simons and Clevers, 2011). Such an analysis, however, has not yet been achieved for continuously growing adult tissues, but is essential if we wish to understand the architecture of adult organs. The retinas of lower vertebrates grow throughout life from retinal stem cells (RSCs) and retinal progenitor cells (RPCs) at the rim of the retina, called the ciliary marginal zone (CMZ). Here, we show that RSCs reside in a niche at the extreme periphery of the CMZ and divide asymmetrically along a radial (peripheral to central) axis, leaving one daughter in the peripheral RSC niche and the other more central where it becomes an RPC. We also show that RPCs of the CMZ have clonal sizes and compositions that are statistically similar to progenitor cells of the embryonic retina and fit the same stochastic model of proliferation. These results link embryonic and postembryonic cell behaviour, and help to explain the constancy of tissue architecture that has been generated over a lifetime.

## Abstract (precis)

**Summary:** A quantitative study of cell proliferation and fate choice in the zebrafish retina - a continuously growing neural tissue - reveals key features of late retinal neurogenesis.

## INTRODUCTION

The retinas of fish and amphibians grow continuously throughout much of life by adding rings of new cells at the periphery, the so-called ciliary marginal zone (CMZ; Johns, 1977; Straznicky and Gaze, 1971). The CMZ provides an excellent model for studying the proliferation in continuously growing tissues. In the past decades, the CMZ of lower vertebrates has been under intense investigation with respect to the genes, signalling pathways and transcriptional profiles that drive stem cells at the peripheral edge of the CMZ through proliferation and differentiation into the adult neurons that emerge at the central edge of the CMZ (Borday et al., 2012; Cerveny et al., 2012; Moshiri et al., 2004).

Early clonal analysis of the CMZ (Wetts et al., 1989) showed it to consist of two types of proliferative cells: those that give rise to large growing clones that stay in contact with the CMZ and those that give rise to small differentiated clones that lose contact with the CMZ. The former were assumed to be retinal stem cells (RSCs) and the latter, retinal progenitor cells (RPCs) (Wetts et al., 1989). More recent work in medaka fish using indelible genetic markers shows that there are indeed dedicated RSCs in the CMZ, which give rise to arched continuous stripes (ArCoSs) containing all retinal cell types (Centanin et al., 2011). The fact that these ArCoSs are of constant width suggests that RSCs undergo invariant asymmetric divisions (Centanin et al., 2014).

Molecular studies reveal that cells from peripheral to central CMZ have different expression profiles of transcription factors, signalling molecules and cell cycle genes (Agathocleous and Harris, 2009; Cerveny et al., 2012; Harris and Perron, 1998; Johns, 1977; Raymond et al., 2006). Genes that are expressed at earlier developmental stages of retinogenesis are expressed in more peripheral regions of the CMZ, whereas genes that are expressed later in development are expressed more centrally, suggesting that what happens in time through embryonic development is recapitulated spatially in the CMZ (Harris and Perron, 1998). Analysis of markers combined with BrdU uptake and label retention show that the cells located at the extreme peripheral edge of the CMZ and presumed to be RSCs divide slowly, whereas cells in the central part of the CMZ divide more rapidly (Ohnuma et al., 1999; Perron et al., 1998; Xue and Harris, 2012).

To appreciate the relationship between embryonic and postembryonic development in quantitative detail, it makes sense to investigate the proliferative characteristics of CMZ cells in light of what has recently been learned about retinal proliferation during embryogenesis. In zebrafish, the cells that give rise to the optic vesicle divide slowly between 14 and 22 hours post fertilization (hpf). Then, a wave of rapid proliferation starting from the central region of the optic cup starts to spread outwards (He et al., 2012; Hu and Easter, 1999). By 72 hpf, this wave is at the very edge of the retina. It leaves in its wake cells that have exited the cell cycle and differentiated into functional retinal neurons and glia. In front is the rim of undifferentiated and dividing cells at the periphery of the retina, which defines the CMZ.

Live-imaging studies show that when embryonic RPCs first arise in this wave, they initially divide a few times symmetrically and proliferatively (PP), they then enter a short stochastic period in which they undergo PP, PD [a mode in which one daughter differentiates (D) while the other remains proliferative] or DD (a symmetric differentiative) division. This is followed by a final stochastic period in which the probability of a DD division becomes high and most cells quickly exit the cell cycle (He et al., 2012). These early RPCs thus produce clones of variable size with a mean of about 12 differentiated cells per initial RPC. This provides a simple quantitative framework for asking whether postembryonic RPCs of the CMZ behave in a statistically similar manner to embryonic RPCs.

The clonal analysis and imaging studies in this paper show that RSCs reside at the extreme peripheral edge of the CMZ, and that as the retina develops, they divide increasingly asymmetrically in the radial orientation. This axis of cell division leaves one daughter in the stem cell niche where it remains an RSC and one that is pushed centrally, where it becomes an RPC. We also show that the fate behaviour of RPCs of the CMZ is statistically similar to embryonic RPCs. These results provide an explanation as to why the retinas of fish and amphibians can grow throughout a lifetime to produce a neural tissue with a similar cellular architecture, from the embryonically generated centre to the adult periphery.

## RESULTS

### CMZ consists of RSCs and RPCs with distinct potency

**Fig. 1.**
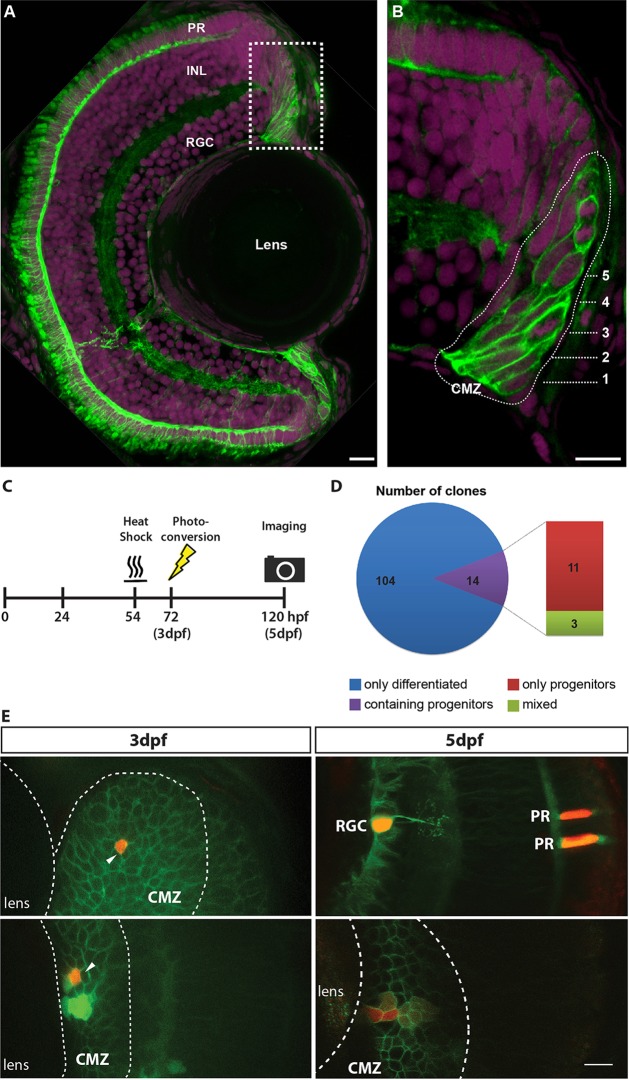
**Short-term clonal analysis.** Rx2 functions as a marker for the CMZ of the zebrafish retina. (A) A maximum intensity projection of a transverse 12 µm section of a 5 dpf zebrafish retina, showing the expression of the transgenic reporter Tg(Rx2:GFPcaax). Rx2^+^ cells (green) are found in the CMZ as well as in two cell types of the neural retina, photoreceptors (PRs) and Müller glia. (B) High-magnification 1 µm section from a region of the CMZ (box in A), revealing that both RSCs (rings 1-2) and RPCs (rings 3-5) are Rx2^+^. Numbers indicate rings. Nuclei are labelled with DAPI (magenta). INL, inner nuclear layer; RGC, retinal ganglion cell layer. (C) Experimental procedure for labelling CMZ cells and capturing the short-term clonal composition. (D) Composition of short-term CMZ clones reveals the distinct proliferative fates in the CMZ population. (E) A single CMZ cell is photoconverted to red at 3 dpf (top left, indicated by arrow) and the resultant terminated clone at 5 dpf (top right, red) is shown with identified cell fates (RGC, retinal ganglion cell; PR, photoreceptors). Another example of a single CMZ cell photoconverted at 3 dpf (bottom left, indicated by arrow) and the resultant maintained clone observed at 5 dpf (bottom right, red). Note the dilution of the label in cells further from the lens, which is suggestive of more rapid division. In E, all Rx2^+^ cells have green membranes whereas the MAZe:Kaede cells have green cytoplasm that becomes red upon photoconversion. Scale bars: 10 µm.

To resolve how individual cells in the CMZ behave in the postembryonic retina, we labelled single cells in 72 hpf fish, which is when the central retina development is complete and proliferation is restricted to the CMZ. We used the MAZe-Kaede system (He et al., 2012) in transgenic fish that carry the membrane-localized progenitor (and Müller glial) marker Rx2:GFPcaax, which was functionally characterized as labelling the CMZ cell population, including neural stem cells (Fig. 1A,B; Chuang and Raymond, 2001; Heermann et al., 2015; Reinhardt et al., 2015). At 54 hpf, a defined heat shock was applied to trigger Cre-mediated recombination in a random subset of cells, driving the expression of the transcriptional activator Gal4:VP16, which then activates expression of the cytoplasmic photoconvertible protein Kaede (Ando et al., 2002) from an upstream activating sequence (UAS) promoter (Collins et al., 2010). At this stage, single GFP-labelled cells in the CMZ were randomly selected for photoconversion to red fluorescence. We found that red fluorescence after photoconversion provided a durable signal for lineage tracing for up to 5 dpf (Fig. 1E).

This assay revealed two populations of CMZ cells with surprisingly distinct proliferation or differentiation behaviour. Of the CMZ cells that were photoconverted at 72 hpf, 88% (104/118) produced fully differentiated clones consisting of retinal neurons or glia cells by 120 hpf (Fig. 1D,E top panel). By contrast, 9% (11/118) of labelled cells generated clones in which all the progenitor cells remained in the CMZ (Fig. 1D,E bottom panel), arranged in a stripe-like structure on the eye. The remaining 3% (3/118) of clones had a mixture of cells that remained in the CMZ and cells that had differentiated and left the CMZ. This bimodality of proliferative characteristics in the CMZ is similar, although not numerically comparable, to what was previously shown in the CMZ for a different species using a different methodology (Wetts et al., 1989), and suggests that we are looking at RSCs and RPCs.

### RSCs reside in the most peripheral edge of the CMZ

**Fig. 2.**
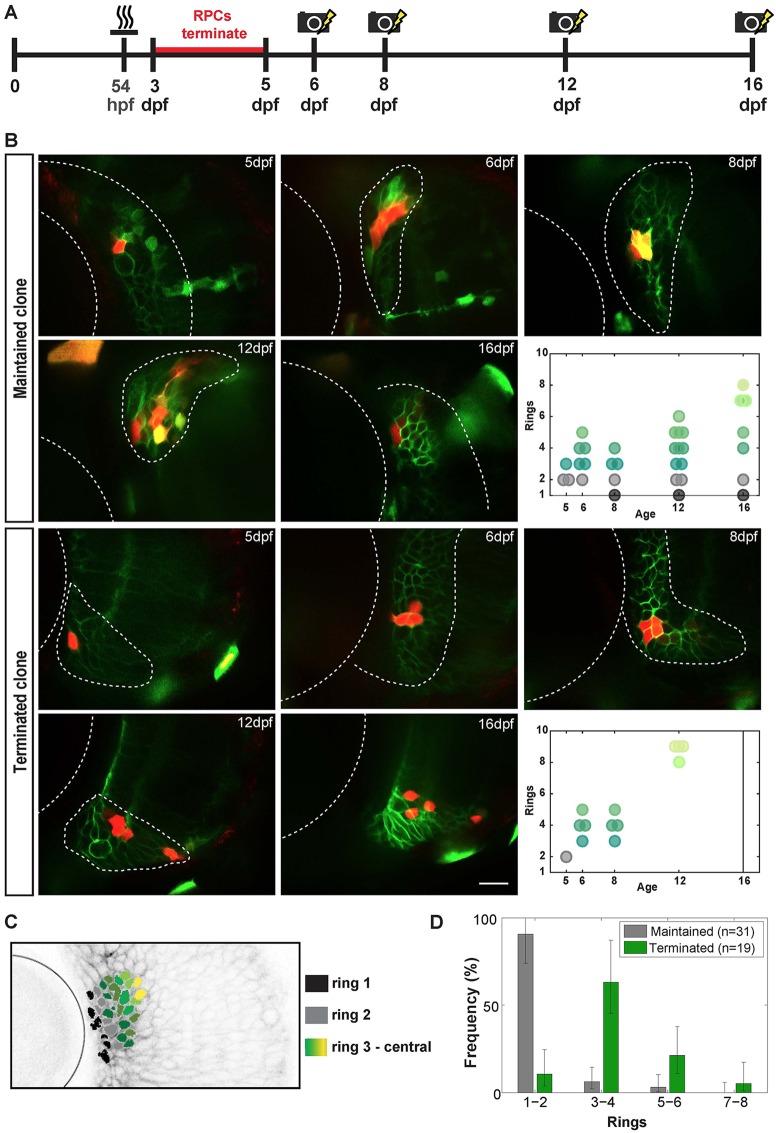
**Long-term polyclonal analysis.** (A) Experimental procedure for labelling 5 dpf CMZ clones and following the maintaining CMZ polyclones at subsequent time points. (B) Photoconverted CMZ cells (red) at 5, 6, 8, 12 and 16 dpf, with the top two rows showing a maintained polyclone and bottom two rows a terminated polyclone at 16 dpf. Rx2^+^ cells have green membranes whereas the MAZe:Kaede cells have green cytoplasm that becomes red upon photoconversion. Yellow areas indicate colocalization of signals. Scale bar: 20 µm. The final panels in each series show the clonal geometry of each of these two clones. Black circles indicate ring 1; grey circles, ring 2; dark green to light green, rings 3-8. (C) Frontal view of an Rx2^+^ retina, with the cell ring position coded in colour (as shown in key). (D) Distribution of the position of the most peripheral cells in clones on the last observed day for all maintained clones (*n*=31) and the corresponding value for all terminated clones before termination (*n*=19). Error bars represents one sigma confidence interval of Poisson distribution.

The low percentage of RSC-derived clones, together with the lack of knowledge of the exact location within the CMZ of the photoconverted cells in the above experiment, led us to adapt the MAZe-Kaede system further so that we could study the RSCs with more precision (Fig. 2A). To label more RSCs, we heat shocked the fish at 54 hpf, but instead of photoconverting at 3 dpf, we left the retina growing for 2 more days and applied the initial photoconversion at 5 dpf. As the vast majority of RPC clones induced at 3 dpf terminate within 2 days, we reasoned that the clones that remained in the CMZ at 5 dpf were likely to contain at least one stem cell. We then tried to photoconvert all the Kaede-positive cells in a connected cell patch in the CMZ at 5 dpf, in effect labelling a polyclone that consists of multiple Rx2-positive cells in the CMZ. We then imaged the photoconverted polyclones over an extended period of time (up to 16 dpf, see Materials and Methods).

Among the 50 polyclones that were followed, 31 continued to retain cells in the CMZ by 16 dpf (*n*=14) or up to the time of death of the fish if earlier (*n*=17, 10 of which died on 12 dpf, and 7 died on 16 dpf), whereas 19 clones became fully differentiated during postembryonic retinal development (Fig. S1). Although it is possible that a maintained polyclone is not equivalent to a polyclone that contains an RSC, to find which cells in our polyclones were most likely to be the RSCs, we looked at the spatial distribution of the photoconverted cells. In a 3D image of the CMZ clone, the stripe-like structure was rotated to an orientation where the lens was facing the viewer, and the surface of the eye at the position of the clone stripe was parallel to the viewing plane (Fig. 2C). If a cell in the clone was within the annulus or ring of CMZ cells that were closest to the lens, it was defined to be in ‘ring 1’ (see also Fig. 1B). The cells that were one cell diameter further away from the lens were deemed to be in ‘ring 2’, etc. Thus, the lower the ring number of a cell, the more peripheral that cell is in the CMZ. This provided a metric of the spatial distribution of cells within the clones with respect to a central-peripheral (radial) axis in the CMZ at any observed time point. This is shown for a single maintained and a single terminated clone in Fig. 2B,C, respectively (see Fig. S1 for all polyclones). Taking into account the spatial arrangement of cells in a clone, it became immediately evident that the maintained clones have their most peripheral cells in ring 1 or 2, whereas the terminated clones detach from these most peripheral rings before termination (Fig. 2D). This positional distribution of maintained and terminated clones is not only observed in the long-term polyclones, but also in single clones traced from 5 to 7 dpf in older larvae (Fig. S2). Thus, we conclude that rings 1 and 2 of the CMZ contain most or all of the RSCs and we therefore suggest that these rings are within the RSC niche, whereas outer rings contain the RPCs.

### RSC divisions become increasingly asymmetric

**Fig. 3.**
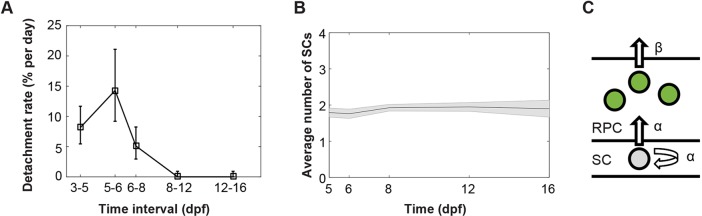
**Stabilization of CMZ clones is due to asymmetrical RSC division.** (A) The rate of clone detachment events plotted as a function of age. Detachment rate is defined as the number of detachment cases (clones move away from rings 1-2 in a specific period of time) divided by the total number of cases surviving up to that time point, divided by the number of days in the time period. (B) Plot showing the average number of ring 1+ring 2 cells in the maintained clones as a function of age. Note that this number remains relatively constant in the maintained clones, indicating potential asymmetrical division of RSCs. (C) Model of RSC and RPC division, with α and β indicating the division rate of RSCs and RPCs, respectively.

It has been proposed that each RSC in the CMZ of larval medaka fish divides asymmetrically to produce one RSC and one RPC to generate an ArCoS that stays in touch with the CMZ (Centanin et al., 2011). Such asymmetric divisions would also assure that the CMZ maintains a constant population of RSCs and provides a natural explanation for the relatively constant width of ArCoSs (Centanin et al., 2014). However, because the space at the extreme edge of the CMZ is strictly limited, it is possible that at early stages of development, there is competition for this niche and that in some cases, both daughters of an RSC might end up within the niche whereas, in others, neither daughter will. As our protocol is designed to label RSCs in polyclones, the fact that a third of the polyclones become detached from the RSC niche suggests that, although there might be a strong bias toward asymmetry, not all RSC divisions are asymmetric at 5 dpf. However, by following polyclones further in time, we find that the rate of clonal detachment from the niche drops significantly and that by 8 dpf, no further detachment of clones from rings 1-2 was observed (Fig. 3A). The higher detachment rate at early time points could be due to the fact that the CMZ has not been fully stabilized and/or that the polyclones do not contain any RSCs by 8 dpf. The fact that the detachment rate drops to near zero indicates that the RSCs in the stem cell niche at the extreme periphery of the CMZ divide in a radial orientation that leads to asymmetric fate outcomes, with one cell always remaining in the niche. A prediction of this asymmetry, and of the radial geometry seen here and in ArCoS clones, is that the number of RSCs per clone should remain constant (Centanin et al., 2014). When we followed the number of RSCs in rings 1-2 of maintained clones for 16 dpf, we found that this was indeed the case (Fig. 3B). Fig. 3C shows a simple model of RSC and RPC divisions that are compatible with these data.

### Orientations of RSC and RPC cell divisions in the CMZ

**Fig. 4.**
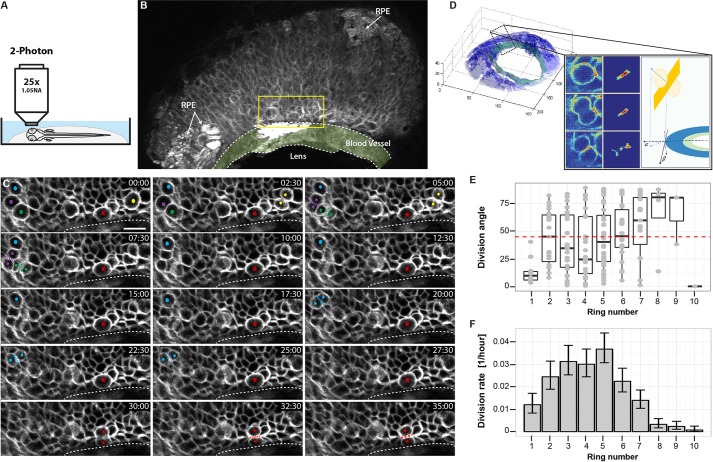
**Live imaging of cell division in the CMZ.** (A) Schematic illustration of the two-photon imaging set-up. (B) 3D projection of one movie frame (*t*=69, *z*=16.5 µm), showing the CMZ region imaged. Arrows indicate areas of retinal pigment epithelium (RPE). Yellow rectangle represents the area shown in the following panels. The ring blood vessel is shaded light green. (C) Selected frames of a 4D time-lapse movie showing several cell divisions in a variety of orientation planes. Dividing cells are labelled with a coloured dot. A presumptive stem cell, dividing parallel to the edge of the retina close to the blood vessel, is labelled with a red asterisk. Each frame represents a maximum intensity projection (six frames, *z*=4.5 µm). Elapsed time shown in min:s. Dotted line indicates the edge of the blood vessel. Scale bar: 10 µm. (D) Computation of division angle. The 3D image stack is segmented into retina (blue) and blood vessel (green). Inset panel shows how the pixels belonging to the divisions are detected by analysing the difference between two consecutive or temporally close frames (depending on the time resolution of the imaging) and finding large contiguous volumes of statistically significant pixels in the difference image, followed by a manual validation step (see supplemental Materials and Methods). Once the divisions are detected, the normal of the division plane (yellow) is estimated and projected in the CMZ plane and the angle between the projected normal and the tangent to the CMZ circle at the closest point is computed. (E) Box plot of division angles detected in each ring of cells in the CMZ. Ring 1 cells divide more strictly axially compared with the random distribution in higher rings. Boxes span the first to the third quartile. Whiskers extend from the lowest value still within 1.5 interquartile range (IQR) of the lower quartile to the highest value still within 1.5 IQR of the upper quartile. (F) Estimated division rate in each ring of cells in the CMZ. Division rate is defined as the number of divisions observed divided by the number of cells imaged in the corresponding ring. Error bars represent one sigma confidence interval of Poisson distribution.

If RSCs of the zebrafish CMZ divide asymmetrically with one daughter remaining as an RSC in the stem cell niche and the other daughter leaving the niche, one could expect that cell divisions of RSCs should be oriented along the radial axis. In this case, we should see evidence of oriented cell divisions in the most peripheral part of the CMZ. To investigate the distribution of cell division orientations within the CMZ, we used two-photon confocal microscopy over several hours using the Tg(Rx2:caaxGFP) line (Fig. 4A). We captured more than 150 cell divisions in two 4D time-lapse movies of 5 dpf larvae (Fig. 4B,C; Movie 1). We noted that, as in the embryonic retina (Das et al., 2003), the vast majority of these divisions take place at the apical surface, and are parallel to this surface (see also Fig. 1B and Fig. S3), i.e. both daughter cells remain in contact with the apical surface. Although there might be small deviations along the apicobasal axis, we focused on the orientation of cell division as a two-dimensional issue; i.e. within the plane of the apical surface of the CMZ. Here, the orientation of cell division can vary from radial, which is defined as orthogonal to the local tangent at the circumference of the CMZ, to circumferential, which is parallel to this tangent (Fig. 4D). For each observed cell division, we then calculated the distance to the edge of the CMZ and the orientation of the division (Fig. 4E,F and supplemental Materials and Methods). Significantly, we found that the most peripheral cells (row 1) divide predominantly radially, whereas divisions of more central CMZ cells are unconstrained in their orientation (Fig. 4E). This significant bias of RSCs to divide radially would tend to keep one daughter close to the peripheral edge, next to the ring of blood vessels that is forming at this time (Fig. 4B, Kitambi et al., 2009), where it could remain in an RSC niche and push the other daughter out of the niche and towards a more central RPC fate.

### RPCs of the CMZ recapitulate the developmental history of embryonic RPCs

**Fig. 5.**
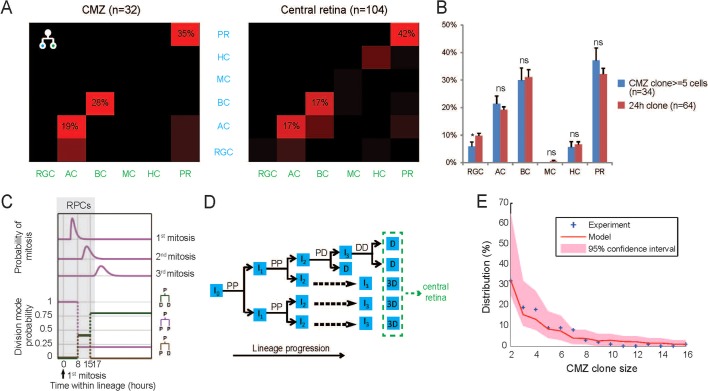
**RPCs recapitulate the developmental history of central retina progenitors.** (A) The frequency of fate combinations of sister cells in two-cell clones is comparable in the CMZ and the central retina. Colour in the matrix is normalized to the ratio of dominant fate combinations. Brightest red colour indicates the most common composition, and black means the fate combination does not exist. (B) Clonal composition of large CMZ clones (≥5 cells) resembles the composition of central retina clones induced at 24 hpf. None of the cell types except for RGCs shows a significant difference in the percentage of the clone (α=5%). (C) Schematic showing the theoretical model used to simulate the RPC clone size. Time-dependent mitosis and division mode probability in central retina development are plotted (adapted from He et al., 2012), with the CMZ progenitors randomly selected from the pool of progenitors in the first 17 h within the lineage, indicated in the purple box. (D) An example of the RPC clone in the CMZ recapitulating the age distribution within a prototypical cell lineage in a central retina progenitor. I_0_, initial progenitor; I_1_, primary progenitor; I_2_, secondary progenitor; I_3_, tertiary progenitor, all residing in the CMZ region (green); D, differentiated cells residing in the central retina (blue). (E) Fit between model predictions (red line with shaded pink region showing 95% plausible intervals due to finite sampling) and size distribution (blue crosses) of RPC clones. In this figure, data shown in the right panel of A, the red bars in B and the model shown in C have all been extracted from He et al. (2012).

Our recent work in the zebrafish suggests that embryonic RPCs are equipotent in terms of their proliferative and fate potential and that they behave stochastically according to highly constrained intrinsic probabilities (He et al., 2012). The distributions of clone size and fate composition of embryonic RPCs are characteristics of these probabilities. Obvious questions therefore arise about the RPCs that are generated in the CMZ. Do these postembryonic RPCs follow the same statistical rules as the embryonic RPCs? To answer these questions, we looked in detail at clones generated by RPCs. By the end of the short-term clonal analysis at 5 dpf, we identified the five basic neuronal types – retinal ganglion cells (RGCs), amacrine cells (ACs), bipolar cells (BPs), horizontal cells (HCs) and photoreceptors (PRs) – in all of the photoconverted clones, and compared them with the lineages reconstructed from live imaging in the central retina during embryonic development (Fig. 5A,B and Fig. S4; He et al., 2012). There was a striking similarity in the distribution of cell types in clones generated from embryonic RPCs and from RPCs in the CMZ. In the CMZ, two-cell clones from a terminal division had a strong preference to specific fate combinations: the clones composed of PR, BC and AC pairs were over-represented, which is consistent with the pattern observed in the embryonic retina (Fig. 5A). Similarly, the cell fate combinations in three-cell clones was also in agreement, with RGCs and two PRs being the prominent type. In the central retina, 93% of the three-cell clones with two PRs contained either an AC or an RGC, whereas the ratio was 94% in the CMZ. Furthermore, the percentage of ACs, BC, HCs and PRs in clones larger than 5 cells was similar to those originating from 24 hpf progenitors in the central retina (Fig. 5B). We also photoconverted single RPCs that gave rise to terminated clones at 5 dpf and analysed these clones at 7 dpf. Although this data set was more difficult to obtain for technical reasons, these 5 dpf RPC clones also showed similar compositions to the embryonic clones assessed by He et al. (2012) and of course the 3 dpf RPC clones (Table S1).

### Proportions and clone sizes

We next focused on the clone size of the RPC lineages. A prototypical clone of 12 cells would be generated by four rounds of mitoses (Fig. 5D): two rounds of PP divisions that turn the initial RPC (I_0_) into two primary progenitors (I_1_) and then four secondary progenitors (I_2_), followed by one round of a mixture of PP, PD and DD divisions that gives four tertiary progenitors (I_3_) and four differentiated cells, and finally the round of DD divisions that terminate the clone (He et al., 2012). In reality, because of the stochastic nature of some of these division modes, few if any clones follow this exact scheme. Yet such a scheme representing average proliferative behaviour is useful for approximating the distribution of different-staged RPCs in the CMZ. As we excluded single clones in the analysis and only focused on the behaviour of RPCs with proliferation potential, we additionally assumed that RPCs can reside in the CMZ for a limited time (17 h in the case of this model based on the 3 dpf data) after their first division (Fig. 5C) before being pushed into the central retina and forced to differentiate. When we initialized the RPC pool in the CMZ by using a mixture of many cell lineages that originated from equipotent I_0_ RPCs (like the typical lineage in Fig. 5D), randomly selected one of the descendant RPCs in the lineage and allowed it to fully differentiate following the probabilistic model in Fig. 5C, the experimental clone size distribution of the RPC cells fell well within the 95% confidence interval of the predicted distribution from the model (Fig. 5E).

In the 24 hpf embryonic retina, a prototypical I_0_ RPC generates a clone of 12 neurons, which is the outcome of 11 divisions. In the CMZ, we have shown that an RSC typically makes an asymmetrical division to give rise to one copy of itself and one early (I_0_) RPC. Thus, if these early RPCs of the CMZ are equivalent to embryonic RPCs in terms of their proliferative potential, we might expect a ratio of 1 RSC division to 11 RPC divisions. How does this prediction match the assumption that RSCs reside in ring 1 and RPCs reside in the more central rings? We found that 12 cells in ring 1 went through cytokinesis in the same time interval as 153 cells in ring 2 and higher, yielding a ratio of 1:13. This is broadly consistent with the prediction and might be even closer if, as seems likely from our clonal imaging analyses, a small fraction of cells in ring 2 were RSCs. It is also clear from these time-lapse recordings that the proportion of cells that can be seen to go through cytokinesis during the defined period of these movies depends on the distance from the periphery. The proliferation rate was low in the most peripheral annulus corresponding to ring 1, and it then increased in the middle of the CMZ and decreased again toward the central CMZ (Fig. 4F). This agrees qualitatively with our hypothesis that, from the periphery to the centre, the CMZ consists of RSCs that divide slowly, young RPCs that divide quickly, and finally, old RPCs that are leaving the cell cycle and beginning to differentiate. This too, is in accordance with what has been found within the embryonic retina, where RSCs divide slowly (Hu and Easter, 1999), early RPCs divide symmetrically in a PP mode and late RPCs slow down their cell cycles and differentiate (He et al., 2012).

## DISCUSSION

Using quantitative clonal strategies, we confirmed that RSCs are located at the peripheral extremity of the CMZ, which functions as a stem cell niche (Raymond et al., 2006; Xue and Harris, 2012), where they divide mainly in the radial orientation promoting asymmetric fate outcome and ensuring a constant production of RPCs. We also show that the same simple model (using the same parameters) that was shown to describe the clonal evolution in embryonic RPCs (He et al., 2012) fits the proliferative statistics of RPCs in the CMZ, suggesting the functional equivalency of the embryonic and CMZ progenitor cells.

The location of RSCs at the extreme periphery of the CMZ is consistent with previous studies based on molecular markers and label retention (Ahmad et al., 2004; Johns, 1977; Ohnuma et al., 2002; Xue and Harris, 2012). The position of RSCs in the CMZ, with access to the apical epithelial surface and close association with the ring blood vessel, is a feature that is common to other neurogenic niches in the vertebrate brain (Raymond et al., 2006) and supports the idea that there is a growth factor-dependent stem cell niche at the peripheral edge of the CMZ (Xue and Harris, 2012). This study also demonstrated that RSCs divide in a persistently asymmetrical manner to produce one RSC and one early stage RPC, as is strongly suggested by the constant thickness of ArCoSs (Centanin et al., 2014, 2011).

The multipotency of clones derived from RPCs in the *Xenopus* CMZ (Wetts et al., 1989) suggested that adult RPCs and embryonic RPCs share some fundamental properties. This notion was reinforced by later studies, using a variety of differentiation and cell cycle markers, showing that the CMZ spatially recapitulates, from the peripheral to the central, the temporal progression of embryonic retinal development (Johns, 1977; Ohnuma et al., 2002; Raymond et al., 2006). Here, we show that CMZ-derived RPCs are not significantly different in terms of their statistical proliferation patterns to embryonic RPCs, suggesting that they are functionally equivalent cell types, which helps to explain the constancy of retinal tissue architecture in zebrafish from the centre to the periphery. We did not see any Müller glia in our 3-5 dpf terminated clones. This is not unexpected because of the low percentage of Müller glia in the retina and our small sample size, yet it raises the question of whether the central Müller glia contribute to the cellular architecture of the peripheral retina or whether it all arises from the CMZ. Although our work here does not address this question, Centanin et al. (2011) showed that the ArCoS clones contain all retinal neurons and Müller glia, and thickly label all cells within their width, suggesting that the cellular architecture of the retina arises from clones that originate in the CMZ. Our paper builds on their work by showing that RPCs share the same proliferative potential and fate behaviour as embryonic RPCs, which offers a quantitative explanation for the homogeneity of retinal architecture.

The key difference between the embryonic generation of the central retina and the postembryonic generation of the peripheral retina, which continues throughout much of life in frogs and fish, is that the latter is fuelled by a population of self-renewing RSCs in the CMZ. During the early formation of the optic vesicle in zebrafish, the cell cycle is very slow and then, at about 24 hpf, a wave of proliferation spreads from the centre of the retina reaching the periphery by 72 hpf (He et al., 2012). The peripheral rim that remains proliferative is the initial CMZ and at its extreme periphery is the stem cell niche.

In many homeostatic adult epithelial tissues, stem cells can frequently commit to terminal differentiation, and the loss of these stem cells is compensated by the multiplication of neighbouring stem cells (Simons and Clevers, 2011). In such homeostatic self-renewing tissues, where stem cell duplication happens with the same probability as termination, the tissue is eventually taken over by clones that dominate through neutral competition (Vogel et al., 1969). In contrast to such scenarios, indelible genetic markers used for the long-term tracking of clones originating in the CMZ of medaka fish (Centanin et al., 2011) show that retinal clones derived from stem cells do not take over, but rather form long thin ArCoSs, comprising all types of retinal cells that stretch from the central retina to the still-growing CMZ. The fact that such ArCoSs rarely terminate and rarely gain width strongly suggests the absence of such neutral competition and suggests instead that the RSCs generating these clones divide strictly asymmetrically (Centanin et al., 2014). Our polyclonal analysis at a cellular level of resolution supports these observations by showing that RSC division is asymmetric in terms of fate. We also find that these asymmetric divisions tend to be radially oriented. One unifying explanation for these two observations is that RSC competence is ensured by factors located at the extreme edge of the CMZ, near the ring blood vessel that lies between the lens and the retina (Kitambi et al., 2009). Clone terminations were observed in our young but not older fish, suggesting that the CMZ is stabilized during the first few days of postembryonic development.

Asymmetric divisions along particular axes have been seen to be important during the development of both plants and animals. For example, in the developing nervous system, asymmetric divisions along the apicobasal axis, are thought to lead to the unequal inheritance of intrinsic determinants such as Numb (Roegiers and Jan, 2004). In many systems, however, the oriented division of stem cells can lead to one cell remaining with a niche that provides short-range signals, such as growth factors, which maintains the ‘stemness’ of the cells that remain within the niche (Januschke and Näthke, 2014). This seems to be the case in the CMZ of the zebrafish, where the critical orientation is not apicobasal but radial, which helps ensure that one daughter cell remains at the extreme periphery of the retina while the other is forced out. This raises many interesting questions. For example, do these radial divisions lead to the asymmetric segregation of intrinsic factors that lead to differential fates, or is it just that one daughter remains in the stem cell niche while the other moves out? If the latter is the case, one would also like to know how this orientation is achieved, whether the blood vessel is an important component of the niche and whether the same signals that regulate proliferation in adult mammalian stem cell niches (Li and Clevers, 2010) are used in this system. These questions must await further investigation of this fascinating proliferative neural tissue.

## MATERIALS AND METHODS

### Animals and transgenic lines

Zebrafish were maintained and bred at 28.5°C. Embryos were staged in hpf before 72 hpf and in dpf afterwards. Embryos were treated with 0.003% phenylthiourea (PTU, Sigma) at 8 hpf to delay pigmentation, and anaesthetized with 0.035-0.040% MS-222 (Sigma) prior to live imaging. The Tg(UAS:Kaede) (Scott and Baier, 2009), the MAZe (Collins et al., 2010) and the Tg(Rx2:GFPcaax) (Heermann et al., 2015) transgenic lines have been described previously. The double transgenic line Tg(MAZe,Rx2:GFPcaax) was generated by crossing the MAZe with the Tg(Rx2:GFPcaax) line. For photoconversion experiments, the Tg(MAZe,Rx2:GFPcaax) line was crossed with the Tg(UAS:Kaede) line. The nlsRFP of the original MAZe line caused retinal cells to die. Therefore, as in Jie et al. (2012), we photoconverted Kaede-expressing cells that showed no nlsRFP expression.

### Heat shock and photoconversion

For clonal analysis, Tg(MAZe,Rx2:GFPcaax,UAS:Kaede) embryos were heat shocked either at 10 hpf or 54 hpf for 5-20 min at 37°C, and allowed to recover at 28.5°C. At 72 hpf, the embryos were screened on a Nikon Eclipse TE2000 upright fluorescent microscope with a 40× water-immersion objective for Rx2-positive retinas with Kaede-expressing cells. The selected embryos were then embedded in 3% methylcellulose and transferred to a spinning-disc microscope (Perkin Elmer UltraVIEW VoX) equipped for photoconversion coupled with an Olympus 40× water immersion objective (NA 1.3), where photoconversion was performed on green Kaede-expressing cells. For short-term analysis, at 3 dpf or 5 dpf, single cells in the CMZ region (defined by Rx2 membrane marker) were randomly targeted by applying a 5 s train of 405 nm laser pulses until the cell turned red. For long-term clonal analysis, the same photoconverting procedure was applied initially at 5 dpf but on all the cells in a connected Kaede cell patch in the CMZ region. The photoconversion was then repeated on the converted cells that still remain in the CMZ at subsequent time points, such as 6, 8, 12, and 16 dpf or until the death of the fish.

### Confocal image acquisition and analysis

For short-term clonal analysis, 5 dpf or 7 dpf embryos were collected, anaesthetized and embedded in 3% methylcellulose with the proper orientation. For transverse sections, 5 dpf fish retinas were fixed in 4% PFA, cryosectioned, stained with DAPI and mounted using Fluorsave (Calbiochem). Retina clones and sections were imaged using an Olympus 60× silicon oil objective (NA=1.35) on an inverted laser-scanning confocal microscope (LSCM; Olympus FV1000), because the resolution of the image acquired on a LSCM is higher than one from the photo-conversion equipped spinning disc. All the images were acquired using comparable settings (1024×1024 resolution, 10 µs/pixel scanning speed, 1-1.2 µm optical section). For long-term clonal analysis, the images were acquired using the spinning disc confocal microscope straight after photoconversion (1000×1000 resolution, 250 ms/frame exposure time, 1 µm optical section). Image analysis was performed using Volocity software (Improvision).

### Two-photon imaging acquisition and analysis

Tg(Rx2:GFPcaax) embryos were selected at 5 dpf and embedded in 1% low-temperature-melting agarose in a standard 90 mm Petri dish. Following addition of the imaging medium (0.3× Danieau's solution, 1× PTU, 0.8× MS222), the embedded embryos were placed in a 28.5°C heated chamber and imaged with a LaVision BioTec TriM ScopeII two-photon microscope, using a 25× (NA 1.05) water-immersion objective. The movies were acquired using comparable settings (0.75-1 µm optical section, 2.5-3 min interval, for 3-5 h). The 3*d+t* data were analysed using Fiji and MATLAB (MathWorks) software. Data analysis consists of the following three stages: detecting the position of the CMZ edge, identifying the divisions and the orientation of the division planes and finally, computing the relevant statistics for each division (angle of division and approximate distance from the CMZ edge). For further information on image analysis, see Computation of division angles and the relevant statistics in the supplemental Materials and Methods.

### Numerical simulation of the RPC clone size

The RPC clone size distribution predicted by the stochastic models in the text was based on the previously described simulation of clone size in the central retina (He et al., 2012). Two key points were inherited from the original model: (1) the cell cycles of the central retina progenitor cells follow a shifted gamma distribution, with a refractory period of 4 h, mean of 6 h and width of 1 h; (2) the transition from symmetrical proliferation (PP) followed by asymmetrical (PD) and then terminal (DD) divisions were simplified to an internal clock starting from the first mitosis, with the probability initiated by pure PP division in the period 0-8 h, to 20% PP, 40% PD and 40% DD during the period 8-15 h and then, finally, to 20% PP and 80% DD after 15 h. The model was adapted to simulate the RPC proliferation based on the assumptions discussed in the text. The mathematical simulation was implemented using Monte Carlo methods in a custom program written in MATLAB (MathWorks).

## References

[DEV133314C1] Agathocleous M. and Harris W. A. (2009). From progenitors to differentiated cells in the vertebrate retina. *Annu. Rev. Cell Dev. Biol.* 25, 45-69. 10.1146/annurev.cellbio.042308.113259 19575661

[DEV133314C2] Ahmad I., Das A. V., James J., Bhattacharya S. and Zhao X. (2004). Neural stem cells in the mammalian eye: types and regulation. *Semin. Cell Dev. Biol.* 15, 53-62. 10.1016/j.semcdb.2003.09.003 15036208

[DEV133314C3] Ando R., Hama H., Yamamoto-Hino M., Mizuno H. and Miyawaki A. (2002). An optical marker based on the UV-induced green-to-red photoconversion of a fluorescent protein. *Proc. Natl. Acad. Sci. USA* 99, 12651-12656. 10.1073/pnas.202320599 12271129 PMC130515

[DEV133314C4] Borday C., Cabochette P., Parain K., Mazurier N., Janssens S., Tran H. T., Sekkali B., Bronchain O., Vleminckx K., Locker M. et al. (2012). Antagonistic cross-regulation between Wnt and Hedgehog signalling pathways controls post-embryonic retinal proliferation. *Development* 139, 3499-3509. 10.1242/dev.079582 22899850

[DEV133314C5] Centanin L., Hoeckendorf B. and Wittbrodt J. (2011). Fate restriction and multipotency in retinal stem cells. *Cell Stem Cell* 9, 553-562. 10.1016/j.stem.2011.11.004 22136930

[DEV133314C6] Centanin L., Ander J.-J., Hoeckendorf B., Lust K., Kellner T., Kraemer I., Urbany C., Hasel E., Harris W. A., Simons B. D. et al. (2014). Exclusive multipotency and preferential asymmetric divisions in post-embryonic neural stem cells of the fish retina. *Development* 141, 3472-3482. 10.1242/dev.109892 25142461 PMC4197724

[DEV133314C7] Cerveny K. L., Varga M. and Wilson S. W. (2012). Continued growth and circuit building in the anamniote visual system. *Dev. Neurobiol.* 72, 328-345. 10.1002/dneu.20917 21563317

[DEV133314C8] Chuang J. C. and Raymond P. A. (2001). Zebrafish genes rx1 and rx2 help define the region of forebrain that gives rise to retina. *Dev. Biol.* 231, 13-30. 10.1006/dbio.2000.0125 11180949

[DEV133314C9] Collins R. T., Linker C. and Lewis J. (2010). MAZe: a tool for mosaic analysis of gene function in zebrafish. *Nat. Methods* 7, 219-223. 10.1038/nmeth.1423 20139970

[DEV133314C10] Das T., Payer B., Cayouette M. and Harris W. A. (2003). In vivo time-lapse imaging of cell divisions during neurogenesis in the developing zebrafish retina. *Neuron* 37, 597-609. 10.1016/S0896-6273(03)00066-7 12597858

[DEV133314C11] Harris W. A. and Perron M. (1998). Molecular recapitulation: the growth of the vertebrate retina. *Int. J. Dev. Biol.* 42, 299-304. 9654012

[DEV133314C12] He J., Zhang G., Almeida A. D., Cayouette M., Simons B. D. and Harris W. A. (2012). How variable clones build an invariant retina. *Neuron* 75, 786-798. 10.1016/j.neuron.2012.06.033 22958820 PMC3485567

[DEV133314C13] Heermann S., Schütz L., Lemke S., Krieglstein K. and Wittbrodt J. (2015). Eye morphogenesis driven by epithelial flow into the optic cup facilitated by modulation of bone morphogenetic protein. *eLife* 4, 373 10.7554/eLife.05216 PMC4337729 25719386

[DEV133314C14] Hu M. and Easter S. S. (1999). Retinal neurogenesis: the formation of the initial central patch of postmitotic cells. *Dev. Biol.* 207, 309-321. 10.1006/dbio.1998.9031 10068465

[DEV133314C15] Januschke J. and Näthke I. (2014). Stem cell decisions: a twist of fate or a niche market? *Semin. Cell Dev. Biol.* 34, 116-123. 10.1016/j.semcdb.2014.02.014 24613913 PMC4169664

[DEV133314C16] Johns P. R. (1977). Growth of the adult goldfish eye. III. Source of the new retinal cells. *J. Comp. Neurol.* 176, 343-357. 10.1002/cne.901760304 915042

[DEV133314C17] Kitambi S. S., McCulloch K. J., Peterson R. T. and Malicki J. J. (2009). Small molecule screen for compounds that affect vascular development in the zebrafish retina. *Mech. Dev.* 126, 464-477. 10.1016/j.mod.2009.01.002 19445054 PMC2775549

[DEV133314C18] Li L. and Clevers H. (2010). Coexistence of quiescent and active adult stem cells in mammals. *Science* 327, 542-545. 10.1126/science.1180794 20110496 PMC4105182

[DEV133314C19] Moshiri A., Close J. and Reh T. A. (2004). Retinal stem cells and regeneration. *Int. J. Dev. Biol.* 48, 1003-1014. 10.1387/ijdb.041870am 15558491

[DEV133314C20] Ohnuma S.-i., Philpott A., Wang K., Holt C. E. and Harris W. A. (1999). p27Xic1, a Cdk inhibitor, promotes the determination of glial cells in Xenopus retina. *Cell* 99, 499-510. 10.1016/S0092-8674(00)81538-X 10589678

[DEV133314C21] Ohnuma S., Hopper S., Wang K. C., Philpott A. and Harris W. A. (2002). Co-ordinating retinal histogenesis: early cell cycle exit enhances early cell fate determination in the Xenopus retina. *Development* 129, 2435-2446. 11973275 10.1242/dev.129.10.2435

[DEV133314C22] Perron M., Kanekar S., Vetter M. L. and Harris W. A. (1998). The genetic sequence of retinal development in the ciliary margin of the Xenopus eye. *Dev. Biol.* 199, 185-200. 10.1006/dbio.1998.8939 9698439

[DEV133314C23] Raymond P. A., Barthel L. K., Bernardos R. L. and Perkowski J. J. (2006). Molecular characterization of retinal stem cells and their niches in adult zebrafish. *BMC Dev. Biol.* 6, 36 10.1186/1471-213X-6-36 16872490 PMC1564002

[DEV133314C24] Reinhardt R., Centanin L., Tavhelidse T., Inoue D., Wittbrodt B., Concordet J.-P., Martinez-Morales J. R. and Wittbrodt J. (2015). Sox2, Tlx, Gli3, and Her9 converge on Rx2 to define retinal stem cells in vivo. *EMBO J.* 34, 1572-1588. 10.15252/embj.201490706 25908840 PMC4474531

[DEV133314C25] Roegiers F. and Jan Y. N. (2004). Asymmetric cell division. *Curr. Opin. Cell Biol.* 16, 195-205. 10.1016/j.ceb.2004.02.010 15196564

[DEV133314C26] Scott E. K. and Baier H. (2009). The cellular architecture of the larval zebrafish tectum, as revealed by gal4 enhancer trap lines. *Front. Neural Circuits* 3, 13 10.3389/neuro.04.013.2009 19862330 PMC2763897

[DEV133314C27] Simons B. D. and Clevers H. (2011). Strategies for homeostatic stem cell self-renewal in adult tissues. *Cell* 145, 851-862. 10.1016/j.cell.2011.05.033 21663791

[DEV133314C28] Straznicky K. and Gaze R. M. (1971). The growth of the retina in *Xenopus laevis*: an autoradiographic study. *J. Embryol. Exp. Morphol.* 26, 67-79. 5565078

[DEV133314C29] Vogel H., Niewisch H. and Matioli G. (1969). Stochastic development of stem cells. *J. Theor. Biol.* 22, 249-270. 10.1016/0022-5193(69)90004-6 5783913

[DEV133314C30] Wetts R., Serbedzija G. N. and Fraser S. E. (1989). Cell lineage analysis reveals multipotent precursors in the ciliary margin of the frog retina. *Dev. Biol.* 136, 254-263. 10.1016/0012-1606(89)90146-2 2478403

[DEV133314C31] Xue X. Y. and Harris W. A. (2012). Using myc genes to search for stem cells in the ciliary margin of the Xenopus retina. *Dev. Neurobiol.* 72, 475-490. 10.1002/dneu.20887 21465669

